# VISTA Ligation Reduces Antitumor T-Cell Activity in Pancreatic Cancer

**DOI:** 10.3390/cancers15082326

**Published:** 2023-04-17

**Authors:** David Digomann, Johannes Strack, Max Heiduk, Ioana Plesca, Luise Rupp, Charlotte Reiche, Simone Nicolaus, Carolin Beer, Ulrich Sommer, Marc Schmitz, Marius Distler, Jürgen Weitz, Adrian M. Seifert, Lena Seifert

**Affiliations:** 1 Department of Visceral, Thoracic and Vascular Surgery, Faculty of Medicine and University Hospital Carl Gustav Carus, Technische Universität Dresden, 01307 Dresden, Germany; 2 National Center for Tumor Diseases (NCT/UCC), 01307 Dresden, Germany; 3 German Cancer Research Center (DKFZ), 69120 Heidelberg, Germany; 4 Faculty of Medicine and University Hospital Carl Gustav Carus, Technische Universität Dresden, 01307 Dresden, Germany; 5 Helmholtz-Zentrum Dresden-Rossendorf (HZDR), 01328 Dresden, Germany; 6 Institute of Immunology, Faculty of Medicine Carl Gustav Carus, Technische Universität Dresden, 01307 Dresden, Germany; 7 Institute of Pathology, Faculty of Medicine Carl Gustav Carus, Technische Universität Dresden, 01307 Dresden, Germany; 8 German Cancer Research Center (DKFZ), German Cancer Consortium (DKTK), Partner Site Dresden, 69120 Heidelberg, Germany; 9 Else Kröner Clinician Scientist Professor for “Translational Tumor Immunological Research”, 01307 Dresden, Germany

**Keywords:** pancreatic cancer, VISTA, prognosis, cytokines

## Abstract

**Simple Summary:**

The V-domain Ig suppressor of T-cell activation (VISTA) suppresses T-cell effector function, and has been investigated as an alternative immunotherapeutic target in several cancers. In this study, we found increased VISTA expression in pancreatic tumor cells to be associated with decreased overall survival. Immune cells also expressed VISTA, and interaction with tumors cells further enhanced their VISTA expression. Recombinant VISTA inhibited proinflammatory T-cell function and cytokine production. In a syngeneic orthotopic model, VISTA-blockade resulted in decreased tumor weights. VISTA is a potential target that may be included in immunotherapeutic strategies for the treatment of PDAC.

**Abstract:**

Immunotherapy has shown promising results in multiple solid tumors and hematological malignancies. However, pancreatic ductal adenocarcinoma (PDAC) has been largely refractory to current clinical immunotherapies. The V-domain Ig suppressor of T-cell activation (VISTA) inhibits T-cell effector function and maintains peripheral tolerance. Here, we determine VISTA expression in nontumorous pancreatic (*n* = 5) and PDAC tissue using immunohistochemistry (*n* = 76) and multiplex immunofluorescence staining (*n* = 67). Additionally, VISTA expression on tumor-infiltrating immune cells and matched blood samples (*n* = 13) was measured with multicolor flow cytometry. Further, the effect of recombinant VISTA on T-cell activation was investigated in vitro, and VISTA blockade was tested in an orthotopic PDAC mouse model in vivo. PDAC showed significantly higher VISTA expression compared to that of a nontumorous pancreas. Patients with a high density of VISTA-expressing tumor cells had reduced overall survival. The VISTA expression of CD4^+^ and CD8^+^ T cells was increased after stimulation and particularly after a coculture with tumor cells. We detected a higher level of proinflammatory cytokine (TNFα and IFNγ) expression by CD4^+^ and CD8^+^ T cells, which was reversed with the addition of recombinant VISTA. A VISTA blockade reduced tumor weights in vivo. The VISTA expression of tumor cells has clinical relevance, and its blockade may be a promising immunotherapeutic strategy for PDAC.

## 1. Introduction

Pancreatic ductal adenocarcinoma (PDAC) is the seventh leading global cause of cancer death, with a four-to-fivefold higher incidence in countries with a high human development index (HDI) [1]. Overall, it has a devastating 5-year survival rate of only 11% [2]. Fewer than 20% of patients present with resectable disease at the time of diagnosis [3]. Surgical resection remains the only potential curative treatment for pancreatic cancer. Therefore, multimodal concepts including neoadjuvant therapy for locally advanced or oligometastatic disease are particularly important for enhancing resectability. In addition, tests for early detection and the development of new therapeutic approaches are urgently needed [4]. Immunotherapeutic strategies have significantly improved the overall survival in multiple solid tumors, but had very limited efficacy in PDAC. An immunotherapeutic approach should be considered only in tumors with a high mutational burden, as found in tumors with mismatch repair deficiency (dMMR, <1% of all PDAC) [5,6,7,8,9]. An immune suppressive tumor microenvironment with low levels of neoantigens, constrained levels of effector T cells, altered tumor stroma, and a high number of suppressive myeloid cells prevents a sufficient antitumoral immune response [10,11,12,13,14]. In more than two-thirds of all PDACs, an immune-exhausted environment was associated with reduced overall survival [15]. Higher levels of CD4^+^ or CD8^+^ T cells, on the other hand, are associated with improved survival [16,17]. A gene signature of high cytolytic activity shows an increased expression of immune checkpoints, and the expression of immune checkpoint protein programmed death 1 (PD-1) in regulatory T cells (Tregs) in PDAC-draining lymph nodes and tumor was associated with lymph-node metastasis [18,19]. Overall, the data indicate the high relevance of tumor-infiltrating immune cells in PDAC, and suggest that a suitable immunotherapeutic approach has yet not been found. The cytotoxic T-lymphocyte-associated antigen-4 (CTLA-4) and PD-1 immune checkpoints are negative regulators of T-cell immune function, and the inhibition of these receptors led to an increased activation of the immune system [20]. The V-domain Ig suppressor of T-cell activation (VISTA), also called programmed cell death protein 1 homolog (PD-1H), is a Type 1 transmembrane protein that has molecular similarities with immune checkpoint programmed death ligand 1 (PD-L1) [21]. VISTA acts as a ligand and a receptor [22]. It plays an important role in regulating innate and adaptive immune responses [23]. VISTA expression on antigen-presenting cells (APCs) inhibits T-cell proliferation and cytokine production, while an inhibiting antibody increases T-cell activation. Further, VISTA expression on T cells was mainly responsible for a shift of conventional CD4^+^ T cells to Tregs [22,24]. Studies targeting VISTA have shown promise in inflammatory diseases and cancer. In a mouse model of multiple sclerosis, VISTA inhibition accelerated disease progression. Furthermore, a VISTA knockout mouse model showed the spontaneous development of cutaneous and systemic autoimmune diseases resembling human lupus. The effect was partly reversed using an agonistic VISTA antibody [22,25,26]. An inhibitory VISTA antibody reduced the emergence of tumor-specific Foxp3^+^CD4^+^ Tregs, and suppressed tumor growth in melanoma [27]. Prostate-cancer patients treated with anti-CTLA-4 (ipilimumab) showed an increased expression of PD-L1 and VISTA by immune cells, suggesting that VISTA may be a compensatory inhibitory pathway after ipilimumab treatment. Altogether, these preclinical results have led to a Phase I trial using a combined PD-L1-, PD-L2-, and VISTA-antagonist (CA-170) in lymphoma, and another Phase I trial using an anti-VISTA antibody in patients with advanced solid tumors [28,29,30,31,32,33,34]. Despite the investigations of VISTA in different cancers, the role of VISTA in PDAC is largely unknown. In this study, we investigate the expression patterns and mechanisms of VISTA in PDAC by analyzing paraffin-embedded tumor tissue, freshly isolated immune cells from the tumor, and peripheral blood. Further, we treated T cells cocultured with tumor cells with recombinant VISTA, and orthotopic PDAC mice with a monoclonal VISTA antibody. 

## 2. Results

### 2.1. VISTA Is Expressed in Human PDAC

**Figure 1:**
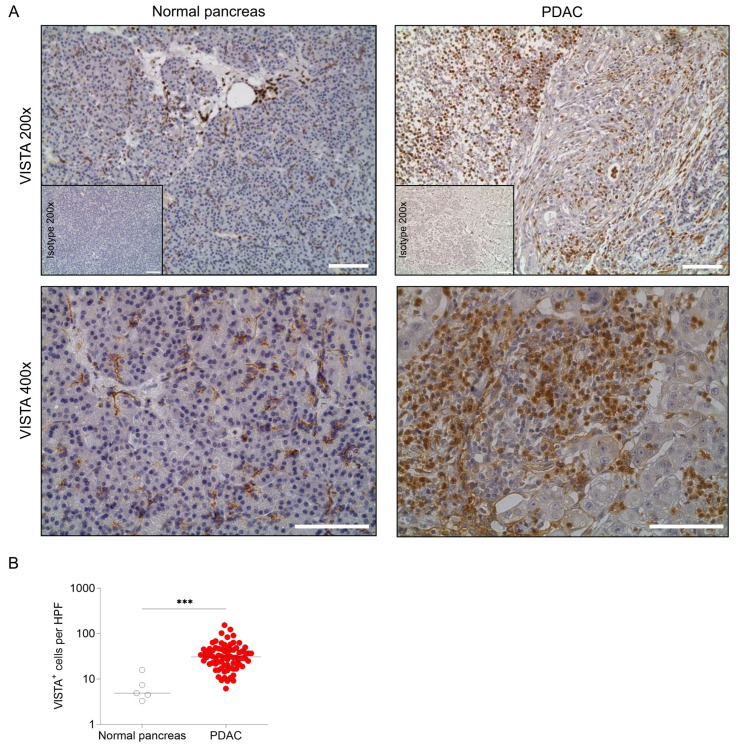
VISTA is expressed in pancreatic ductal adenocarcinoma. (**A**) Representative IHC staining for VISTA in normal pancreatic tissue and pancreatic adenocarcinoma with isotypic control (200× and 400× magnification); scale bar is 100 µm. (**B**) VISTA^+^ cells per high-power field (HPF) in pancreatic adenocarcinoma (*n* = 76) compared to normal pancreatic tissue (*n* = 5; *** *p* < 0.001; Mann–Whitney test).

**Figure 2:**
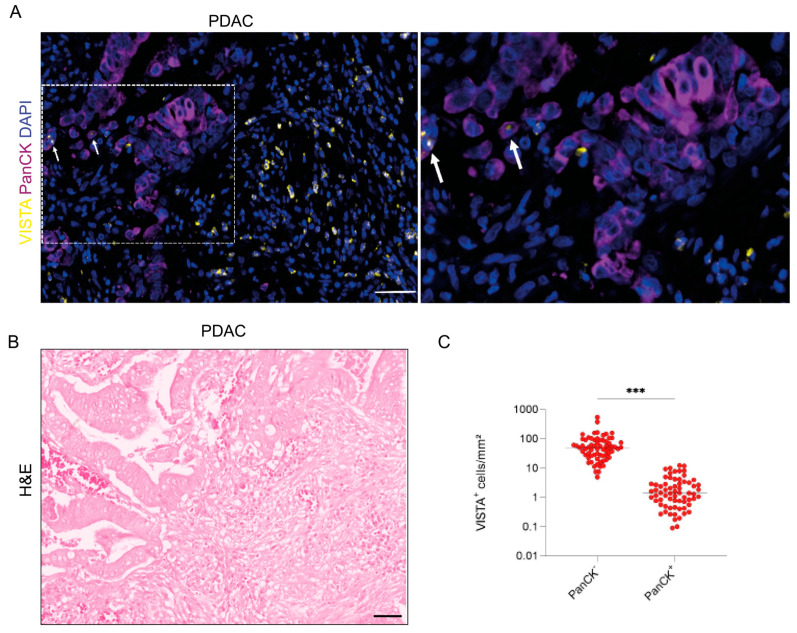
VISTA is expressed by cancer (PanCK^+^) and noncancer (PanCK^−^) cells located at the tumor site. (**A**) Representative multiplex immunofluorescence staining (VISTA in yellow, PanCK in purple, and DAPI in blue) of pancreatic adenocarcinoma with a magnified section. Double positive cells (PanCK^+^/VISTA^+^) cells are marked with arrows; scale bar is 50 µm. (**B**) Hematoxylin and eosin (H&E) stain to isualize the tumor area from (**A**); scale bar is 50 µm. (**C**) Noncancer cells (PanCK^−^) displayed significantly higher VISTA expression compared to that of cancer cells (PanCK^+^; *** *p* < 0.001; Mann–Whitney test).

**Figure 3:**
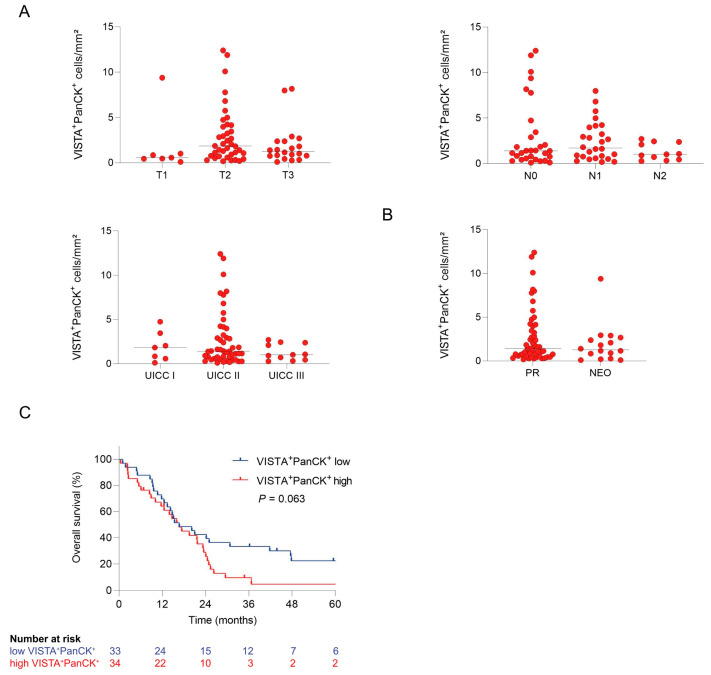
Impact of VISTA expression on clinicopathologic features. (**A**) VISTA expression on PanCK^+^ was not associated with tumor size, lymph-node metastasis, or UICC stage (*n* = 67; Kruskal–Wallis test with Dunn’s statistics). (**B**) Neoadjuvant therapy did not influence VISTA expression (*n* = 67; Mann–Whitney test). (**C**) Survival analysis of PDAC patients according to their VISTA^+^ PanCK^+^ expression (median) shown as Kaplan–Meier curves (*n* = 67; overall survival: *p* = 0.063; log-rank test).

To assess the expression of VISTA in human PDAC, we evaluated 76 sections of tumor tissue and 5 adjacent nontumorous pancreatic tissue samples via immunohistochemistry for VISTA. The normal pancreas samples showed little expression of VISTA. In contrast, VISTA expression was higher in the tumor tissue (Figure 1A,B). Next, we compared the expression of VISTA on cancer (PanCK^+^) and noncancer (PanCK^−^) cells in PDAC patients (*n* = 67) with multiplex immunofluorescence staining. PanCK^-^ cells displayed significantly higher VISTA expression than that of PanCK^+^ cells (Figure 2A–C). However, VISTA expression on PanCK^+^ and PanCK^−^ was not associated with tumor size, lymph-node metastasis, or UICC stage. Further, neoadjuvant treatment did not change VISTA expression on PanCK^+^ or PanCK^−^ (Figure 3A,B and Supplementary Figure S1A,B). Next, we investigated the overall survival of PDAC patients with high or low VISTA expression on PanCK^+^ and PanCK^−^, and no differences between high and low VISTA^+^ PanCK^−^ cells were revealed. However, patients with a high level of VISTA-expressing cancer cells (VISTA^+^ PanCK^+^) showed a trend towards worse overall survival (Figure 3C and Supplementary Figure S1C). An association with other clinicopathologic characteristics was not detected (Supplementary Table S2).

To review our results, the expression of VISTA gene (*VSIR)* was analyzed using RNA expression data deposited in The Cancer Genome Atlas (TCGA) and the Genotype-Tissue Expression (GTEx) database. PDAC patients (*n* = 179) showed higher *VSIR* expression compared to that of normal pancreatic tissue (*n* = 171; Supplementary Figure S2A). Further, no significant differences in *VSIR* expression were found in tumor size, lymph-node infiltration, or tumor UICC stage (eighth edition; Supplementary Figure S2B). When the cohorts were separated into tercentiles, a high level of *VSIR* was associated with better prognosis (Supplementary Figure S2C). This indicated high immune cell infiltration, which is associated with better survival. Therefore, the association between high *VSIR* expression and different immune cell markers was analyzed. A high level of *VSIR* was associated with a high level of leukocyte marker *PTPRC* (CD45) and T-cell marker *TBX21;* no significant difference in *CD3E*, *CD4*, CD8, *FOXP3*, and *GATA3* could be revealed. Moreover, the marker for antigen-presenting cells *HLA-DRA* corresponded with high *VSIR* expression, but no significant association of *VSIR* and the marker for monocyte/macrophage *ITGAM*/*CD14* could be found (Supplementary Figure S2D). Of all the tested immune checkpoint modulators in PDAC, *VSIR* showed the highest expression level among all genes, underlining the importance of VISTA in the immune landscape of PDAC (Supplementary Figure S2E). 

### 2.2. VISTA Inhibits Proinflammatory T-Cell Function and Cytokine Production

**Figure 4:**
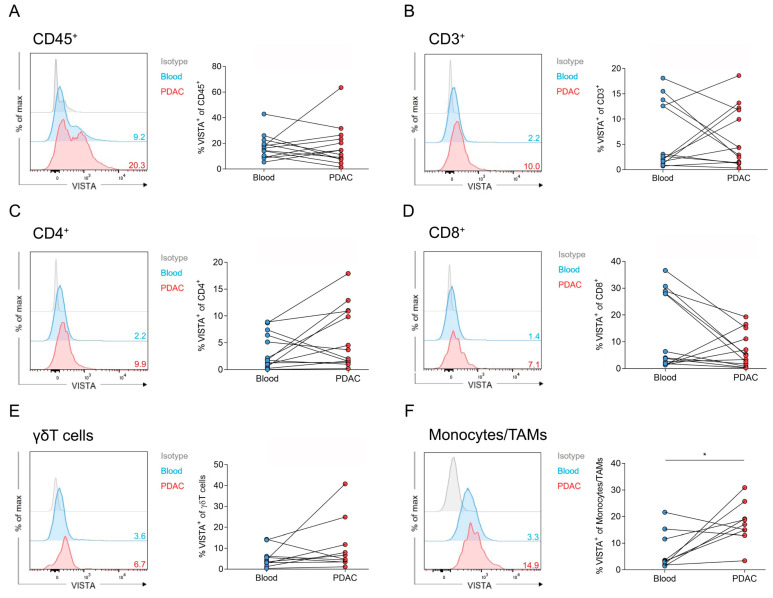
Tumor-infiltrating immune cells highly express VISTA in human PDAC. (**A**–**F**) The expression of VISTA on different tumor-infiltrating immune cells in human PDAC tissue samples was measured and quantified with flow cytometry. Significant differences were found between the blood and tumor in monocytes/TAMs (* *p* = 0.015; paired *t*-test; *n* = 13).

**Figure 5:**
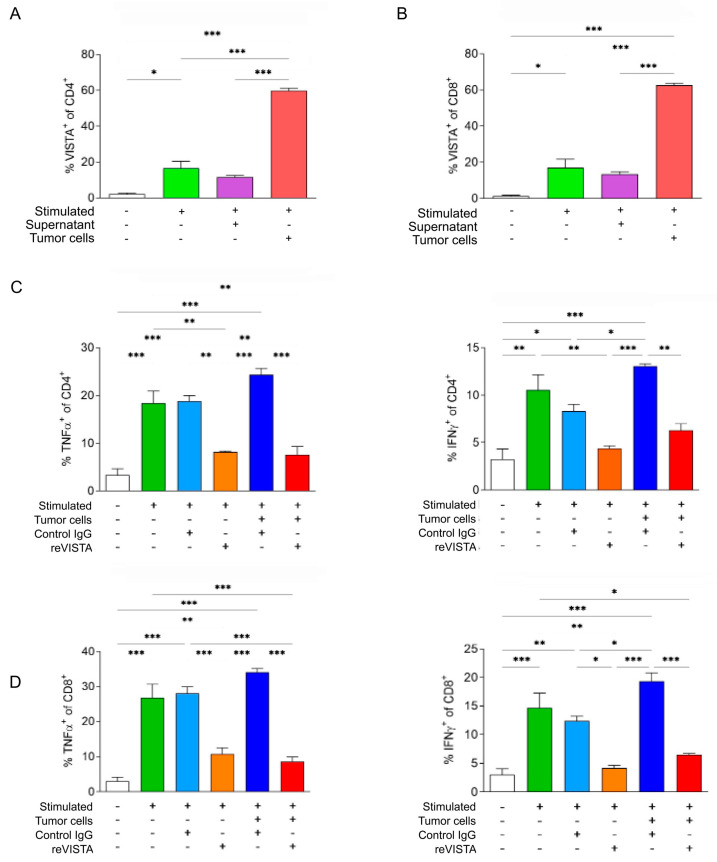
Tumor cells promote VISTA expression on T cells, while VISTA leads to the inhibition of cytokine production. (**A**,**B**) VISTA expression of CD4^+^ and CD8^+^ T cells increased significantly after stimulation and after coculturing with AsPC-1 cells (* *p* < 0.05, *** *p* < 0.001; one-way ANOVA with Tukey statistics). (**C**,**D**) The cytokine production (TNFα and IFNγ) of CD4^+^ and CD8^+^ T cells significantly increased after stimulation (one-way ANOVA with Tukey statistics). Treatment with recombinant VISTA significantly reversed cytokine production in stimulated and AsPC-1-cocultured conditions (* *p* < 0.05, ** *p* < 0.01, *** *p* < 0.001; one-way ANOVA with Tukey statistics).

To further investigate the role of the VISTA expression of tumor-infiltrating immune cells, we performed flow cytometry on immune cells from the tumor specimens and matched freshly obtained blood from PDAC patients (*n* = 13) undergoing surgery. VISTA was highly expressed on tumor-infiltrating immune cells. In addition, different immune subpopulations were investigated regarding their particular VISTA expression. Tumor-infiltrating macrophages showed a significant upregulation of VISTA compared to the corresponding monocytes in the blood, whereas VISTA on different T-cell subsets was expressed at the same level (Figure 4A–F). To further analyze the role and effect of VISTA on immune cells, T cells were cocultured with pancreatic tumor cells or a supernatant from a pancreatic tumor cell culture, and VISTA expression on T cells was determined. After coculturing with tumor cells, CD4^+^ and CD8^+^ T cells built significantly more VISTA, and VISTA expression with the supernatant was similar (Figure 5A,B). To analyze the effect of VISTA on T cells, T cells alone or cocultured with tumor cells were treated with a recombinant VISTA protein, and the proinflammatory cytokine production (TNFα and IFNγ) of CD4^+^ and CD8^+^ T cells was analyzed. Stimulated T cells cocultured with tumor cells showed increased TNFα and IFNγ expression on both cell types, whereas recombinant VISTA protein reversed the stimulation effect, and reduced TNFα and IFNγ expression on both cell populations (Figure 5C,D).

### 2.3. VISTA Blockade Reduces Tumor Growth in an Orthotopic PDAC Mouse Model

**Figure 6:**
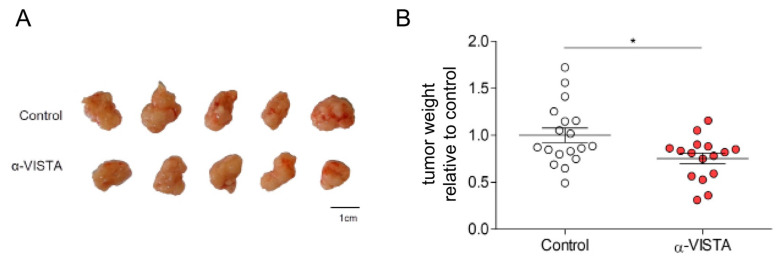
VISTA mAb treatment suppressed tumors in an orthotopic PDAC mouse model. (**A**) Representative tumors of the orthotopic mouse model treated with α-VISTA or the control. (**B**) Tumor weight was significantly reduced when treated with α-VISTA compared to the control (* *p* < 0.05; unpaired *t*-test).

To investigate possible translational applications, an inhibiting monoclonal VISTA antibody was used in an orthotopic PDAC mouse model. Then, 3 days after surgical tumor implantation, a blocking VISTA antibody or control protein was injected subcutaneously. After 15 days, tumors were resected, and tumor weights were determined. The treated animals showed a significantly reduced tumor weight relative to the control (Figure 6A,B). 

## 3. Discussion

Although the 5-year survival of pancreatic-cancer patients has increased to 11% over the last few years, the prognosis is still devastating, and improvement in therapy is urgently needed [2]. Though PD-L1 expression was correlated with prognosis in PDAC, it is rarely expressed in PDAC, and clinical trials targeting PD-L1 showed no promising results [35,36]. Overall, current immunotherapy has not brought the desired success in PDAC, in contrast to other solid tumors, and further therapeutic approaches are necessary [37]. The VISTA protein shows similar structural properties to those of PD-L1, but the VISTA gene had the highest expression among immunomodulators in PDAC. Thus, in this study, we investigated the role of VISTA in PDAC. Our data indicated unfavorable survival in tumors with a high level of VISTA expressed by their cells, but not by tumor-infiltrating immune cells. Supporting data were found on mRNA level. This strengthens the hypothesis that VISTA expression on cancer cells, rather than on tumor-infiltrating immune cells, is a crucial mechanism that compromises survival. This result is consistent with previous findings showing that the VISTA expression of PDAC-infiltrating T cells was not associated with patient outcomes [16]. Furthermore, in human ovarian and endometrial cancers, the VISTA expression on cancer cells suppressed T-cell proliferation and cytokine production in vitro, and anti-VISTA treatment extended the survival of tumor-bearing mice [38]. However, VISTA expression on tumor-infiltrating immune cells also plays an important role in different cancer entities. Particularly, VISTA was described as a potent inhibitory checkpoint on CD68^+^ macrophages when comparing an immunotherapy-sensitive tumor (melanoma, *n* = 44) with an immunotherapy-resistant tumor (pancreatic cancer, *n* = 67) [31]. In prostate cancer, VISTA is a compensatory inhibitory pathway after ipilimumab therapy and could be the reason for treatment failure [28]. Similar results were found in colorectal cancer, for which anti-VISTA treatment could overcome resistance to anti-PD-1/CTLA-4 treatment [39]. Our data confirm that infiltrating immune cells highly express VISTA in human PDAC. At the gene expression level, *VSIR* was associated with a high level of leukocyte marker *CD45*, which is more detailed with T-cell markers *CD3E*, *CD4*, and *TBX21,* and APC marker *HLA-DRA.* Coculturing experiments were performed to further investigate the role of tumor and immune-cell interaction on VISTA expression. We showed a significant upregulation of VISTA after coculturing T cells with tumor cells, which was similar to findings in colorectal cancer [40]. A high level of VISTA inhibited T-cell function and cytokine production in ovarian and endometrial cancer [38]. We showed that VISTA significantly reduces the TNFα and IFNγ expression of CD4^+^ or CD8^+^ T cells cocultured with pancreatic tumor cells. Further, an inhibiting mAB against VISTA in an orthotopic PDAC mouse model led to a reduced weight. This finding is concurrent with previous studies in ovarian, endometrial, and colorectal cancer, and in melanoma, where anti-VISTA treatment suppressed tumor growth and prolonged survival in mouse models [27,38,39]. Further, these results encourage the recently initiated clinical trials with anti-VISTA mAB treatment in patients with advanced solid tumors, including PDAC patients [33,41]. However, using a mouse model comes with several limitations that restrain transferability to humans [42]. Likewise, different pancreatic cancer cell lines show distinct properties that can vary from each other [43]. Moreover, the low number of VISTA^+^ tumor cells has a high effect on falsely labeled cells. To strengthen the results, further validation with different cell lines would be beneficial. Overall, our data support the notion of VISTA as an important player in pancreatic cancer and as a potential new immunotherapeutic target.

## 4. Materials and Methods

Patient samples: Blood samples and tumor specimens were obtained from patients with PDAC who had received surgical treatment at our institution. The patients consented to a protocol approved by the ethics committee of TU Dresden (no. EK446112017). Peripheral blood was assessed before the surgical procedure using EDTA tubes (Sarstedt), and mononuclear cells were separated with density centrifugation over a Biocoll Separating Solution (Merck; threefold centrifugation: 800× *g* at 21 °C for 20 min, 500× *g* at 4 °C for 7 min, and 500× *g* at 4 °C for 7 min). The resected cancer tissue was used for immunohistochemical (IHC) staining, multiplex immunofluorescence (mIF) staining, or flow cytometry. For the flow cytometry, the tissue was dissociated using collagenase Type IV, DNase (both Thermo Fisher, Rockford, IL, USA), and trypsin inhibitor (Sigma-Aldrich, St. Louis, MO, USA) to obtain a single-cell suspension. For IHC and mIF staining, formalin-fixed and paraffin-embedded PDAC tissue was used. The clinicopathological characteristics of PDAC patients are presented in Table 1, Table 2 and Table 3.

Immunohistochemistry and multiplex immunofluorescence: The tissue sections were deparaffinized and rehydrated. Peroxidase was blocked, adding 1% hydrogen peroxide (Carl Roth) for 20 min, followed by a streptavidin and biotin solution (Streptavidin/Biotin Blocking Kit; Vector Laboratories) for 15 min each to block the biotin, biotin receptors, and streptavidin-binding sites. The tissue sections underwent heat antigen retrieval in the microwave (2 × 2.5 min, 540 W; 2 × 5 min, 250 W) and further blocked (5% goat serum, 1% BSA, 1.5 M Tris HCl) for 60 min. The VISTA antibody (1:300; Cell Signaling; #64953) or control (1:300; Cell Signaling, #3900) was applied at 4 °C overnight. The Signal-Stain Boost IHC Detection Reagent (Cell Signaling) was added for 30 min, followed by a DAB Peroxidase Substrate solution (Vector Laboratories) according to the manufacturer’s manual. A hematoxylin solution (Merck KGaA) was regressively used for counterstaining. EVOS^®^ FL Auto Imaging Systems was used for image acquisition. Quantifications were performed by processing 10 high-power fields (HPF; ×20) per slide in a blinded manner via ImageJ. Multiplex immunofluorescence staining was performed as described before [16].

Flow cytometry: Single-cell suspensions were used for flow cytometry as described previously [44]. For intracellular cytokine staining, Brefeldin A (10 µg/m; Thermo Fisher Scientific) was added to the cell culture for 4 h at 37 °C, 5% CO_2_. Then, the surface antigens were stained, followed by cell fixation and permeabilization with the BD Cytofix/Cytoperm Kit and cytokine staining (see Table S1 for the used antibodies). Flow cytometry was conducted using an LSR Fortessa flow cytometer (BD Biosciences). Data were analyzed using FlowJo v10 (Treestar, Ashland, OR, USA).

Coculture and treatment: Human PDAC cell line AsPC-1 (obtained from ATCC) was cultured at 37 °C in 5% CO_2_ in an RPMI medium supplemented with 10% FCS, glucose, 10 mmol/L Hepes, and 1 mmol/L sodium pyruvate. Trypsin was used to detach the cells. Prior to the experiments, cells were tested for mycoplasma contamination, T cells were isolated from the peripheral blood of a healthy donor, and the blood was processed as described in the previous section. Then, the T cells were isolated using CD3 MicroBeads (MiltenyiBiotec). CD3^+^ T cells were further transferred to an anti-CD3 or anti-CD3 plus recombinant VISTA/control protein (R&D Systems, #7126-B7-050)-coated 96-well plate and cultured in a T-cell medium (RPMI 1640, supplemented with 10% FCS and 1% Pen/Strep-PreMix) with additional anti-CD28 (2 µg/mL) for 48 h at 37 °C and 5% CO_2_. The U-shaped-96-well plates (Corning) had been coated with 100 mL of the protein solution (10 µg/mL) for 24 h at 4 °C. Unstimulated, stimulated, or stimulated and VISTA-treated T cells were cocultured for 24 h with or without AsPC-1 cells (1:1) or the supernatant of the AsPC-1 cell. Cell-type differentiation, cytokine quantification, and protein expression were further analyzed using flow cytometry. 

Syngeneic orthotopic pancreatic cancer mouse model: Murine pancreatic cancer cell line TB32048 that had been generated from genetically modified C57BL/6-mice (KrasLSL.G12D/+; p53R172H/+; PdxCretg/+) was used. A cell solution (25 µL PBS (Thermo Fisher Scientific), 25 µL Matrigel^®^ (Growth Factor Reduced, Phenol Red-free; Corning), 1 × 10^5^ TB32048 cells)) was injected into the pancreas of 8-week-old female C57BL/6-mice (Charles River Laboratories, Wilmington, MA, USA). The mice were kept according to ethics protocol TVV 85/2017; abdominal incision and the preparation of the pancreas were performed under isoflurane buprenorphine anesthesia. Then, 3 days after the surgery, the blocking VISTA antibody (200 µg, Biolegend, #143704) or control protein (200 µg, Biolegend, #400902) was injected subcutaneously. On Day 18, the mice were sacrificed according to guideline 2010/63/EU, and the tumors were resected and weighted. Three independent replicates were performed.

TCGA RNA-seq analysis: mRNA expression data from different tumor entities were assessed via TCGA RNA-seg datasets using the cBioPortal for Cancer Genomics [45]. All data were FPKM-normalized. The data from pancreatic cancer (*n* = 176) were reduced to data from pancreatic ductal adenocarcinoma with clinicopathologic characteristics (*n* = 146). 

Statistical analysis: the data are presented as a scatter plot with the median or bar graph ± SEM. The data were analyzed for Gaussian distribution using four different tests (D’Agostino-Pearson, Shapiro–Wilk, Anderson–Darling, and Kolmogorov–Smirnov). Depending on the result, nonparametric or parametric tests were used: the Mann–Whitney test, unpaired *t*-test, paired *t*-test, Kruskal–Wallis test with Dunn’s test, or one-way ANOVA with Tukey’s multiple-comparisons test. For the threshold optimization of overall survival, the median was used as the starting point and empirically tested for improvement via Cox regression. Median was the best separating threshold. R statistical software was used for this approach (version 4.2.0, R Core Team (2022). R is a language and environment for statistical computing (R Foundation for Statistical Computing, Vienna, Austria)). A Kaplan–Meier survival plot with log-rank comparison was used for survival analysis. GraphPad Prism 9.0 (GraphPad Software, La Jolla, CA, USA) was used; *p* ≤ 0.05 was considered significant.

## 5. Conclusions

VISTA is an important player in pancreatic cancer and its expression on tumor cells has clinical relevance. VISTA blockade may be a promising immunotherapeutic strategy for PDAC.

## Figures and Tables

**Table 1:** Clinicopathologic characteristics of the IHC PDAC cohort.

	Years	
**Mean age**	67	
**Age range**	47–80	
	***n* (%)**	
**Sex**		
Female	36	(47.37)
Male	40	(52.63)
**pT Stage**		
1	5	(6.58)
2	45	(59.21)
3	25	(32.89)
4	0	(0)
Unknown	1	(1.32)
**pN Stage**		
0	40	(52.63)
1	27	(35.53)
2	9	(11.84)
**M Stage**		
0	76	(100)
1	0	(0)
**UICC Stage**		
I	27	(35.53)
II	39	(51.32)
III	9	(11.84)
IV	0	(0)
Unknown	1	(1.31)
**Neoadjuvant Treatment**		
Yes	25	(32.89)
No	51	(67.11)

**Table 2:** Clinicopathologic characteristics of the mIF PDAC cohort.

	Years	
**Mean age**	67	
**Age range**	36–79	
		***n* (%)**
**Sex**		
Female	36	(53.73)
Male	31	(46.27)
**pT Stage**		
1	7	(10.45)
2	39	(58.21)
3	20	(29.85)
4	0	(0)
Unknown	1	(1.49)
**pN Stage**		
0	30	(44.78)
1	26	(38.8)
2	11	(16.42)
**M Stage**		
0	66	(98.51)
1	1	(1.49)
**UICC Stage**		
I	7	(10.45)
II	48	(71.64)
III	11	(16.42)
IV	1	(1.49)
**Neoadjuvant Treatment**		
Yes	16	(23.88)
No	51	(76.12)

**Table 3:** Clinicopathologic characteristics of the flow cytometry cohort.

	Years	
**Mean age**	67	
**Age range**	49–83	
		***n* (%)**
**Sex**		
Female	5	(38.5)
Male	8	(61.5)
pT Stage		
1	1	(7.7)
2	2	(15.4)
3	8	(61.5)
4	2	(15.4)
**pN Stage**		
0	5	(38.5)
1	6	(46.1)
2	2	(15.4)
**M Stage**		
0	11	(84.6)
1	2	(15.4)
**UICC Stage**		
I	2	(15.4)
II	7	(53.8)
III	2	(15.4)
IV	2	(15.4)
**Neoadjuvant Treatment**		
Yes	2	(15.4)
No	11	(84.6)

## Data Availability

The data presented in this study are available on request from the corresponding author.

## Supplementary Materials

The following supporting information can be downloaded at: https://www.mdpi.com/article/10.3390/cancers15082326/s1, Figure S1: Impact of VISTA expression on clinicopathologic feature. Figure S2: VSIR expression plays an important role in human pancreatic ductal adenocarcinoma. Table S1: List of antibodies used for flow cytometry. Supplementary Table S2: Clinicopathologic characteristics of mIF PDAC cohort.
