# Supplementary material for: VISTA Ligation Reduces Antitumor T-Cell Activity in Pancreatic Cancer
Source: Cancers (Basel). 2023 Apr 17;15(8):2326. doi: 10.3390/cancers15082326 (PMC10136841; doi:10.3390/cancers15082326)
Supplement: Supplementary file 1 [file cancers-15-02326-s001.zip › cancers-2312993-supplementary.pdf]

Figure S1. Impact of VISTA expression on clinicopathologic feature. A VISTA expression on PanCK$^-$ was not associated with tumor size, lymph node metastasis, or UICC stage (n = 67; Kruskal-Wallis test with Dunn's statistics). **B** Neoadjuvant therapy did not influence VISTA expression (n = 67; Mann-Whitney test). **C** Survival analysis of PDAC patients according to their VISTA$^+$ PanCK$^-$ expression (median) shown as Kaplan Meier curves (n = 67; overall survival: $P = 0.24$; Log-rank test).

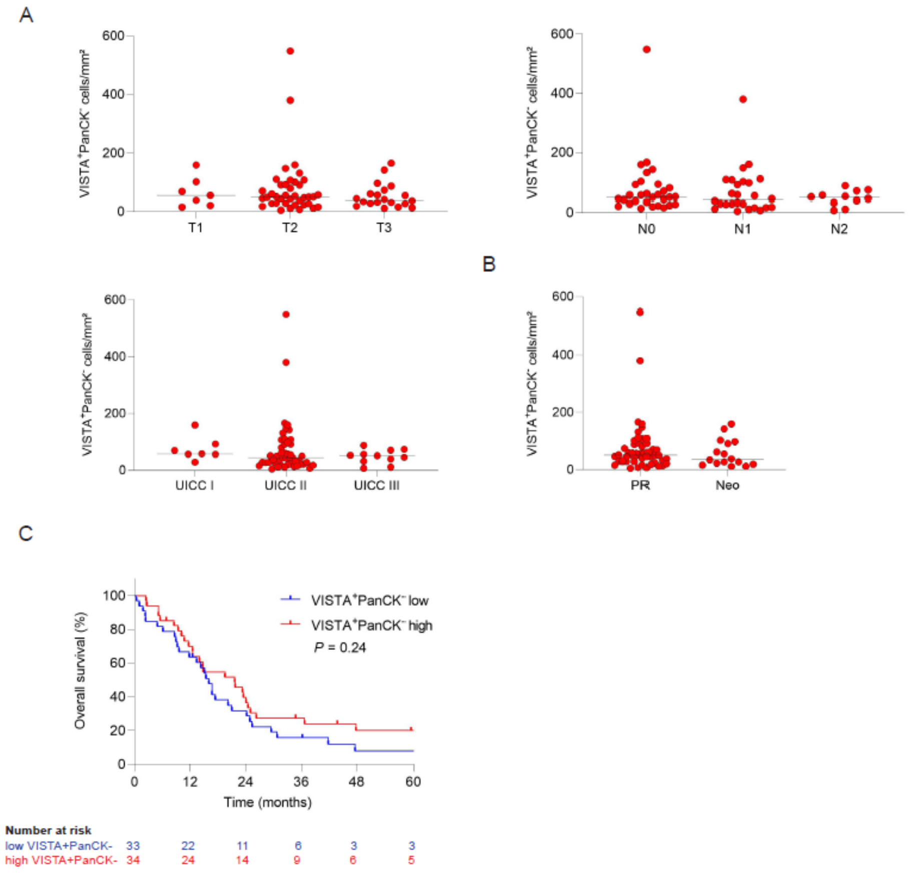

Figure S2. ***VSIR*** **expression plays an important role in human pancreatic ductal adenocarcinoma. A** PDAC patients (n = 179) showed a higher *VSIR* expression compared to normal pancreatic tissue (n = 171; $P < 0.001$; one-way ANOVA). **B** *VSIR* expression was not associated with tumor size, lymph node metastasis, or UICC stage (n = 145; Kruskal-Wallis test with Dunn's statistics or Mann-Whitney test) **C** Kaplan Meier plots of the correlation between *VSIR* mRNA expression (1st and 3rd tercile) and patient survival (n = 91; overall survival: $P = 0.128$; Log-rank test). **D** Association between high and low terciles of *VSIR* mRNA expression and leukocyte marker *PTPRC* (CD45; $P < 0.001$), T cell markers *CD3E* ($P = 0.086$), *CD4* ($P = 0.102$), *CD8A* ($P = 0.099$), *FOXP3* ($P = 0.199$), *GATA3* ($P = 0.184$), *TBX21* ($P < 0.001$), marker of APCs *HLA-DRA* ($P < 0.001$) and macrophages/monocytes *ITGAM*/*CD14* ($P = 0.073$; $P = 0.577$; Mann-Whitney test; n = 96). **E** Boxplot of mRNA expression of immune checkpoints *IDO2*, *ADORA2A*, *TIGIT*, *LAG3*, *PDCD1* (PD-1), *CTLA4*, *PDCD1LG2* (PD-L2), *CD274* (PD-L1), *IDO1*, *HAVCR2* (TIM-3) and *VSIR* (VISTA) in PDAC (n = 144).

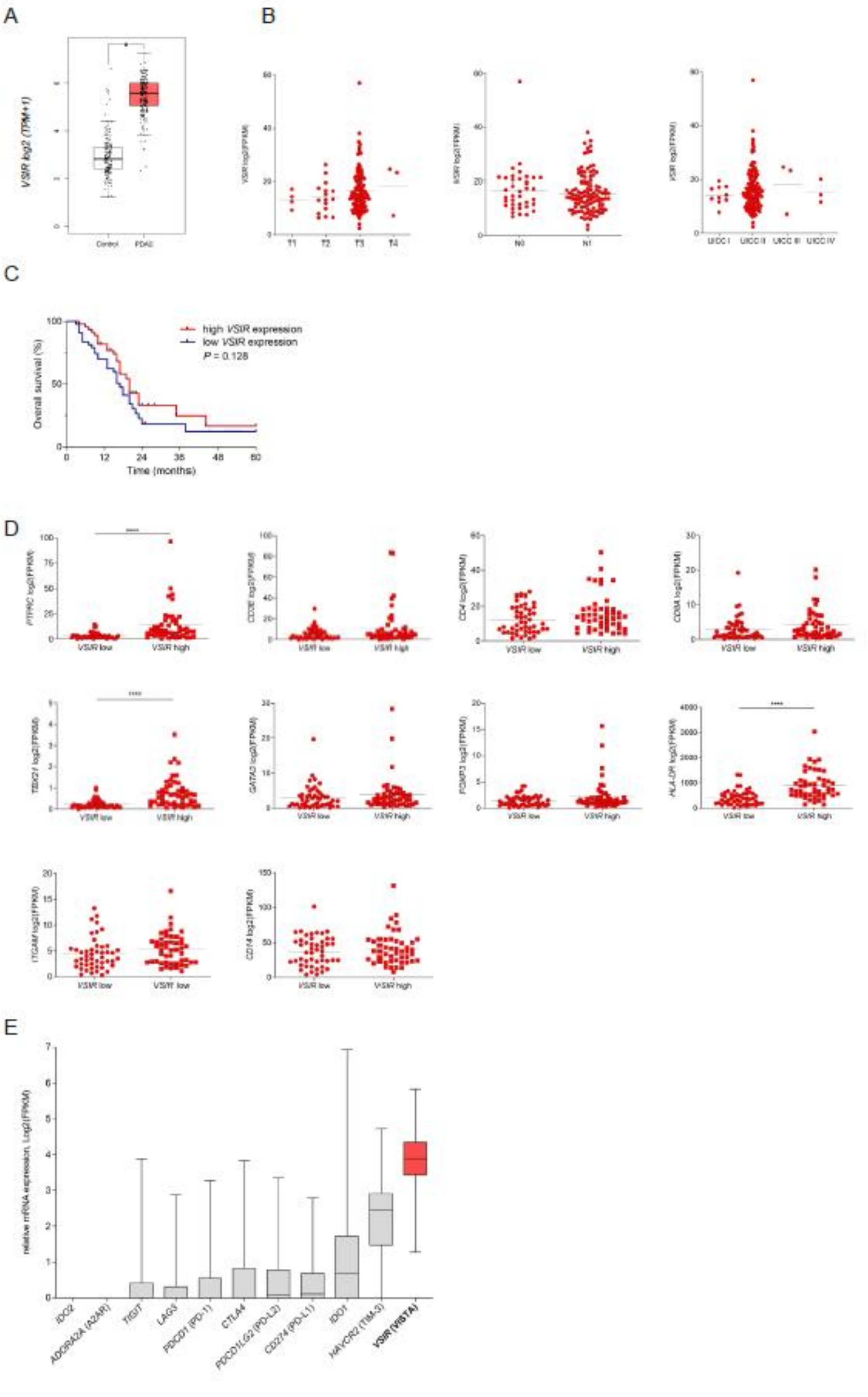

Supplementary Table S1.
List of antibodies used for flow cytometry

Name	Reference
CD11b anti-human AF-700	Biolegend, # 101222
CD14 anti-human V500	BD Biosciences, # 561391
CD19 anti-human PE-Cy7	BD Biosciences. # 557835
CD3 anti-human APC-H7	BD Biosciences, # 560176
CD3 anti-human PE-Cy7	BD Biosciences, # 557851
CD4 anti-human PB	BD Biosciences, # 558116

CD45 anti-human APC	BD Biosciences, # 555485
CD45 anti-human BV605	BD Biosciences, # 564047
CD56 anti-human PE-Cy7	BD Biosciences, # 557747
CD8 anti-human V500	BD Biosciences, # 561618
Hamster IgG	Biolegend, # 400922
IFNγ anti-human BV711	BD Biosciences, # 564793
Mouse IgG1, κ BV711	BD Biosciences, # 563044
Mouse IgG1, κ FITC	Biolegend, # 400110
Mouse IgG2B PE	R&D Systems, # 133303
TCRγδ anti-human PE-Cy7	Biolegend, # 331221
TNFα anti-human FITC	BD Biosciences, # 554512
VISTA anti-human PE	R&D Systems, # 730804

Supplementary Table S2.

Clinicopathologic characteristics of mIF PDAC cohort

		VISTA low		VISTA high	
		**years**		**years**	*P*-value
**Mean age**		67		66	0.82[a]
**Age range**		47 - 77		36 - 79	
		**n (%)**		**n (%)**	*P*-value
**Gender**					
Female	21	(61.76)	15	(42.86)	0.116[b]
Male	13	(38.24)	20	(57.14)	
**pT Stage**					
1	6	(17.65)	1	(2.86)	0.062[b]
2	15	(44.12)	24	(68.57)	
3	11	(32.35)	10	(28.57)	
4	0	(0.00)	0	(0.00)	
Unknown	2	(5.88)	0	(0.00)	
**pN Stage**					
0	16	(47.06)	16	(45.71)	0.492[b]
1	11	(32.35)	15	(42.86)	
2	7	(20.59)	4	(11.43)	
**pM Stage**					
0	34	(100.00)	34	(97.14)	0.321[b]
1	0	(0.00)	1	(2.86)	
**UICC Stage**					
I	3	(8.82)	4	(11.43)	0.567[b]
II	24	(70.59)	26	(74.28)	
III	7	(20.59)	4	(11.43)	
IV	0	(0.00)	1	(2.86)	
**Neoadjuvant Treatment**					

Yes	9	(26.47)	8	(22.86)	0.728[b]
No	25	(73.53)	27	(77.14)	

[a] t-test

[b] Chi-squared test
